# The protective effects of miR-210 modified endothelial progenitor cells released exosomes in hypoxia/reoxygenation injured neurons

**DOI:** 10.1016/j.expneurol.2022.114211

**Published:** 2022-08-24

**Authors:** Sri Meghana Yerrapragada, Harshal Sawant, Shuzhen Chen, Trevor Bihl, Jinju Wang, Ji Chen Bihl

**Affiliations:** a Department of Biomedical Sciences, Joan C Edwards School of Medicine, Marshall University, Huntington, WV 25755, USA; b Department of Pharmacology and Toxicology, Boonshoft School of Medicine, Wright State University, Dayton, OH 45435, USA

**Keywords:** Exosomes, Endothelial progenitor cells (EPCs), miR-210, Neurons, Hypoxia, And reoxygenation (H/R) injury, Oxidative stress

## Abstract

We have previously demonstrated that endothelial progenitor cells (EPCs) provide beneficial effects on ischemic stroke by reducing oxidative stress, which could be through EPCs-released exosomes (EPC-EXs). EXs are emerging as a bioagent for mediating cell-cell communications *via* their carried microRNAs (miR). miR-210 is shown to provide a neuroprotection effect against ischemic stroke. Here, we aimed to determine whether the combination of EPC-EXs and miR-210 would provide an enhanced protective effect on neurons. The hypoxia and reoxygenation (H/R) model were applied to neurons to mimic the ischemic injury of neurons. EPCs were transfected with miR-210 mimic to elevate the level of miR-210 in cells and EPC-EXs (miR210-EPC-EXs). For functional studies, EPC-EXs were co-incubated with H/R-injured neurons, then the cell viability and reactive oxygen species (ROS) production were determined. The results showed 1) H/R induced apoptosis and ROS overproduction in neurons; 2) miR-210 mimic increased the level of miR-210 in both EPCs and EPC-EXs; 3) EPCs cultured in serum-free medium released more exosomes in comparison with cells grown in complete growth media, suggesting serum starving induce the release of EXs; 4) After transfection, EPCs grown in complete media had almost 50 times higher miR-210 level than EPCs had in serum-free media, while the EPCs-EXs isolated from the complete media has lower miR-210 expression than from the serum-free media in a time-dependent manner, suggesting the transfer of miR-210 through EXs; 5) After co-incubation, EPC-EXs and miR210-EPC-EXs were uptaken by neurons, and the miR-210 level in neurons was elevated by miR210-EPC-EXs; 6) miR210-EPC-EXs were more effective in promoting cell viability and decreasing apoptosis and ROS production than EPC-EXs. The present study demonstrated that EPCs-carried miR-210 could be released and transferred to neurons in a time-dependent manner and that miR-210 loading can enhance the protective effects of EPC-EXs on H/R-induced neuron apoptosis, oxidative stress, and decreased viability.

## 1. Introduction

For proper functioning of the brain, neurons require a sufficient amount of glucose and oxygen provided by blood flow. Cerebrovascular disruption in the blood supply to the brain is known as stroke, this can be caused by a blockage in a blood vessel which is referred to as ischemic stroke (Chang and Prabhakaran, 2017). Clot removal is the main goal of ischemic stroke treatment, this can be achieved by medication and mechanical treatments. Tissue Plasminogen Activator (tPA) is considered to be the most effective treatment for stroke (Adams Jr., 1998). In case of tPA treatment does not go well with the patient then mechanical treatments like thrombectomy, angioplasty, and stenting procedures, and stem cells therapy are good options in treating ischemic stroke (Jilani and Siddiqui, 2022; Gurewich, 2016). Previous studies from us and other groups have demonstrated that endothelial progenitor cells (EPCs) play a major therapeutic role in ischemic stroke (Chen et al., 2011; Chen et al., 2013; Li et al., 2021). The underlying mechanism could be attributed to EPC-released exosomes (EXs) (Wu et al., 2016; Ma et al., 2018; Garbuzova-Davis et al., 2017; Terriaca et al., 2021; Wang et al., 2020a).

EXs are extracellular particles, 30–100 nm, released from various cells and found to contain the essential biomolecules such as the mRNAs, microRNAs (miRs), proteins and lipids (Mathivanan et al., 2010; Meldolesi, 2018). Studies have suggested that EXs have the unique function of cellular signaling, wherein they carry information in the form of biomolecules from the parent cell to the recipient cell (Lopez-Verrilli and Court, 2013; Valadi et al., 2007). Along with this, they are involved in the facilitation of immune response, angiogenesis, wound healing, inflammation, and coagulation (Chen et al., 2020; Pegtel and Gould, 2019). According to Wu, Xu, et al., transferring miR-126 to the endothelium through endothelial EXs results in down-regulation on sprouty-related EVH1 domain-containing protein 1 and subsequently promotes RAF/ERK pathway which finally results in an improvement in endothelial function and thereby ameliorates the lung injury (Wu et al., 2018). In 2016, Li X. et al. and Zhang J.et al. explicated that EPC-EXs activate Erk1/2 signaling which stimulates angiogenic activity in endothelial cells (ECs) by which wound healing is accelerated (Zhang et al., 2016). Our previous studies have demonstrated that EPC-EXs protect ECs from hypoxia and reoxygenation (H/R)-induced injury by modulating downstream apoptotic and angiogenesis pathways (Ma et al., 2018; Wang et al., 2020b).

Among the molecules that EXs carry, miRs show the most important role in regulating cellular function. According to Bavelloni et al., miR-210 is strongly linked with the hypoxia pathway, and miR-210 is upregulated in presence of any hypoxia-inducible factors. Hence, miR-210 is considered as master hypoxamir (Chan et al., 2012). The miR-210 influences cell survival by targeting apoptotic genes and it has an antioxidant effect by reducing the production of reactive oxygen species by mitochondria (Chan et al., 2012). Our previous studies have discovered that miR-210 loading could enhance the protective effects of EPC-EXs on H/R injured ECs (Ma et al., 2018), as well as boost the effect of neural progenitor cells (NPCs)-EXs on protecting ECs from angiotensin-II induced injury (Liu et al., 2017). In the present study, we further investigated whether overexpressing of miR-210 could enhance the protective effects of EPC-EXs on H/R-injured neurons.

## 2. Methods

### 2.1. Cell culture

Human EPCs (Celprogen, Torrance, CA) and human neuroblastoma cell lines (SH-SY5Y, ATCC, MD, USA) cell lines were used. EPCs were cultured in EPC complete growth medium with serum and antibiotics in an incubator with 5% CO2 at 37 °C as previously published (Ma et al., 2018; Wang et al., 2020b). The SH-SY5Y neuronal cell lines were grown under standard culture conditions in a culture media with Eagle's Minimum Essential Medium and F-12 K Nutrient Mixture (1:1), fetal bovine serum (10%), and 5% penicillin/streptomycin (P/S) solution (Li et al., 2020).

### 2.2. Generation of miR-210 overexpressing EPC-EXs

The miR-210 mimic was used to generate miR-210 overexpressing EPC-EXs (miR210-EPC-EXs) per our previous publications (Ma et al., 2018; Liu et al., 2017). Briefly, the EPCs were transfected with miR-210-5p mimics or scramble control (Scr) (1 nmol/L, Qiagen) using Dharmafect 1 transfection reagent (Dharmacon) for 48 h in EPC complete growth medium. The complete medium was replaced with a serum-free medium for 48 h to promote the release of EXs. The culture medium was then collected for EXs isolation. EPCs cultured in serum-free medium served as control.

### 2.3. EPC-EX isolation

EPCs were subjected to serum starvation for 48 h to stimulate the release of exosomes from EPCs. Then, the media was collected and centrifuged at 300 xg for 6 min to remove the cell debris and the supernatant was centrifuged at 20,000 xg for 70 min, to pellet microvesicles. Meanwhile, the supernatant from the previous step was collected and centrifuged at 170,000 xg for 90 min to pellet the exosomes per our previous publications (Wang et al., 2020b; Wang et al., 2016). The exosome pellet was suspended with 100 μL sterile-filtered phosphate buffer saline (PBS) for Nanotracking Analysis (NTA) or with the culture media for co-culture functional studies.

### 2.4. Measurement of EX concentration by NTA

The concentration of EXs under different conditions was measured by NS300 instrument (Nanosight, Amesbury, UK) utilizing the light scattering and Brownian motion properties (Wang et al., 2020b; Wang et al., 2016). It can detect the particles of diameter from 10 nm-2 μm and concentration in the range 10^7^–10^9^ particles/mL. The Isolated exosomes are diluted at a concentration of 10^7^–10^9^ particles/mL using filtered PBS and then the sample (700 μL) was loaded into the instrument carefully without creating any air bubbles in the frame. Three videos of particle movements are recorded for each sample at a rate of 30 frames/s and at three different locations. These samples are analyzed using NTA software (version 2.5, Nanosight). The concentration of particles was calculated by considering their dilution factor.

### 2.5. EPC-EX labeling for tracking

To label the EPC-EXs, PKH26, lipophilic-membrane dyes exhibiting red or green fluorescence were used per previous publications (Ma et al., 2018; Wang et al., 2020b). The isolated EXs were incubated with 2 μL of PKH26 (2 × 10^−6^ M, Sigma-Aldrich) in 1 mL PBS for 5 min. To stop the reaction, 1 mL 1% BSA was added and incubated for one minute. The suspension was then ultracentrifuged at 170,000 x g for 90 min to obtain the fluorescent-labeled EXs. The supernatant was discarded, and the pellet was resuspended in a cell culture medium for further coincubation experiments.

### 2.6. Hypoxia and reoxygenation (H/R) injury model for neurons

Under *in-vitro* conditions, ischemic stroke in neurons was induced by exposing the neurons to hypoxia conditions and followed by reoxygenation. Until 80–85% confluency the neurons are grown in a complete SH-SY5Y medium, then they were kept in a hypoxia chamber, with 1% 02, 5% CO2, and 94% N2, for 6 h. After this, the neurons were reoxygenated (37 °C and 5% CO2-standard incubator conditions) for 24 h. During the reoxygenation phase, EPC-EXs was added to neurons for co-culture (Ma et al., 2018; Wang et al., 2020b).

### 2.7. Co-incubation of EPC-EXs with neurons

During the phase of reoxygenation, EPC-EXs or miR210-EPC-EXs (3 × 10^9^ EX particles/mL) were added for co-incubation. The EPC-EXs or miR210-EPC-EXs were resuspended in a complete neuron culture medium and then co-incubated with the neurons for 24 h. Following this, the incorporation rate was measured by analyzing the fluorescent intensity of EPC-EXs (labeled with PKH26) within the cells. After 24 h co-culture, the neurons were collected for various assays to determine the function of EPC-EXs.

### 2.8. PCR analysis of the miR-210 level

The levels of miR-210 in the EPCs, EPC-EXs and neurons after co-incubation were determined by Real-time PCR as we previously published (Ma et al., 2018; Liu et al., 2017). Briefly, the miRNA was extracted from the EPCs, EPC-EXs and neurons by using the TRIzol reagent and the RNA concentration was measured using NanoDrop 2000 Spectrophotometer (Thermo Fisher Scientific). cDNA was synthesized using PrimeScript RT reagent kit (Takara Bio Inc.) following the manufacturer’s instructions. qRT-PCR was carried out using miR-210 specific primers and SYBR Premix Ex Taq kit (Takara Bio Inc.) on a real-time PCR instrument (Bio-Rad). RNA U6 was used as an internal control. The RT primer: 5′- GTC GTA TCC AGT GCA GGG TCC GAG GTA TTC GCA CTG GATACG AC GAC TGT −3′. The primers of miR-210: 5′- CAC GCA GTC GTA TCC AGT GCA GG −3′ (forward); 5′- CCA GTG CAG GGT CCG AGG TA −3′ (reverse). The expression of U6 was used as an endogenous control for each sample. The primers of U6: 5’-CTCGCTTCGGCAGCACA-3′ (forward); 5’-AACGCTTCACGAATTTGCGT-3′ (reverse). The expression of miR-210 was normalized to U6 and calculated using 2 – ΔΔCT method.

### 2.9. Cell apoptosis assay

The cellular apoptotic rate of neurons was determined by using Annexin V/PI apoptosis kit (BD Biosciences). In brief, the cells were resuspended in 100 L of Annexin V binding buffer and labeled with 10 μL of PI and 5 μL of FITC-Annexin V in the dark for 15 min at room temperature. The apoptotic cell was determined as Annexin +/PI – cells and the apoptotic percentage was analyzed using a flow cytometer (Accuri C6).

### 2.10. Intracellular reactive oxygen species (ROS) generation assay

Intracellular ROS generation, an effective method for determining oxidative stress in the cells was determined using dihydroethidium (DHE, Sigma-Aldrich) staining per our previous publications (Ma et al., 2018; Liu et al., 2017). DHE is a superoxide indicator that when oxidized primarily by superoxide results in 2-hydroxyethidium. The neurons were cultured in a 6-well plate and incubated with 10 μM DHE solution (in dark) for 30 min at 37 °C. The cells were then observed under a fluorescence microscope and the percentage of DHE-positive cells was analyzed using the flow cytometer (Accuri C6).

### 2.11. Cell viability measurement

SH-SY5Y neuronal cell viability was measured using MTT-(3- [4, 5-dimethylthiazyol-2yl]-2, 5-diphenyltetrazolium bromide, 5 mg/mL, Sigma) assay. To determine the cell viability of the neurons, SH-SY5Y cell lines were cultured in 96-well plates at a concentration of 2 × 10^3^ cells/ well and injured by subjecting them to hypoxic conditions for 6 h; the cells were then re-oxygenated for 24 h, during the reoxygenation phase the neurons are co-cultured with different EPC-EXs. After co-culturing the cells with exosomes, MTT solution (20 μL) was added and incubated for 4 h at 37 °C. later DMSO (150 μL) was added and incubated for 20 min at 37 °C. On a microplate reader (BioTek, USA) optical density was read at 490 nm. The percent cell viability was calculated by comparing the relative absorbance of treated cells *versus* untreated cells.

### 2.12. Western blot analysis

The proteins of neurons in different co-incubation groups were extracted with cell lysis buffer (Thermo Fisher Scientific). The protein lysates (20 μg) were electrophoresed, transferred onto PVDF membranes and incubated with primary antibody against brain-derived neurotrophic factor (BDNF, 1:500; Abcam), Tropomyosin receptor kinase B (TrkB, 1:1000; Abcam), p-TrkB (1:1000; Abcam), NADPH oxidase 2 (Nox-2, 1:1000; Abcam), Nox-4 (1:1000; Abeam), β-actin (1:4000; Sigma) at 4 °C overnight. Then all membranes were washed and incubated with horseradish-peroxidase-conjugated anti-rabbit or anti-mouse IgG (1:40000; Jackson Immuno Research Lab) for 1 h at room temperature. Blots were developed with enhanced chemiluminescence developing solutions and images were quantified under ImageJ software.

### 2.13. Statistical analysis

All data are presented as mean ± SD. Two group comparison was analyzed by student t-test. Multiple comparisons were analyzed by two-way ANOVA (SPSS version 16.0; SPSS, Chicago, IL, USA and GraphPad Prism version 9.3.0) followed by the Tukey test. For the data with non normality distribution, the non-parametric test (Kruskal-Wallis followed with with paired Mann-Whitney *U* tests) was used. For all tests, a *P*-value <0.05 was considered significant. All the data generated from the experiments were used for the statistical analysis. There are no exclusions.

## 3. Results

### 3.1. Exosomal release pattern and time course of exosome release

**Fig. 1.**
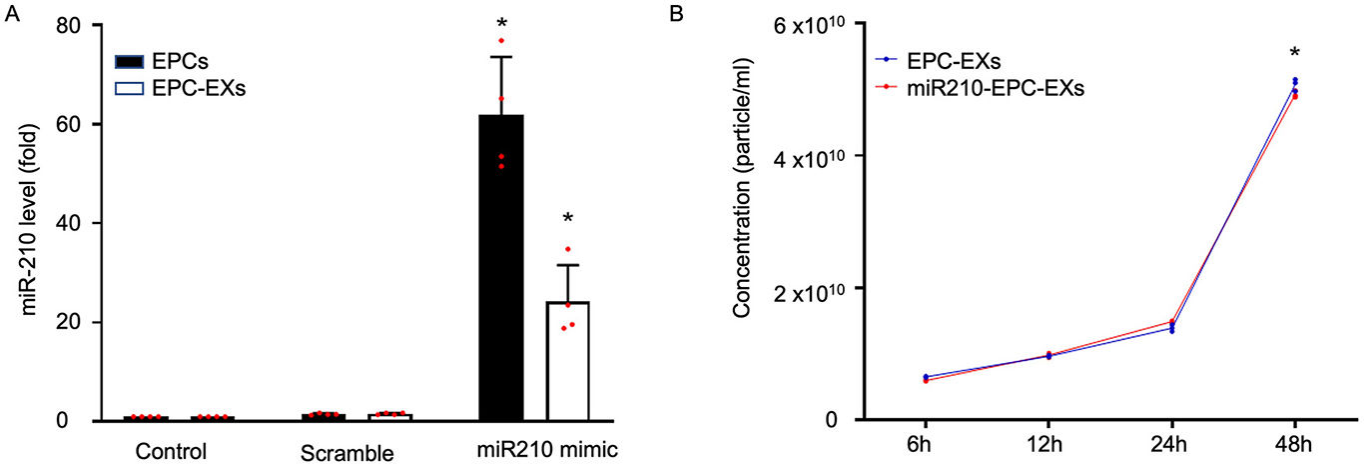
Confirmation of miR-210 levels in EPCs and EPC-EXs after transfection, and the time course release of exosomes. A, The levels of miR-210 in both EPCs and EPC-EXs are significantly increased after transfection of miR-210 mimics. *P < 0.05 *vs*. Control or Scramble. Data represents mean — SD, *n* = 4/group. B, The concentration of EXs released from both EPCs and miR210-EPCs in serum-free media is increased in a time-dependent manner. *P < 0.05 *vs*. 6 h, 12 h or 24 h. Data represents mean ± SD, *n* = 3/group.

To generate miR210-EPC-EXs, EPCs were transfected with miR-210 mimic, and then the EPC-EXs were isolated from the culture media. The miR-210 level in the mimic group was about 50 folds in EPCs, and 25 folds in EPC-EXs higher than the control and scramble group (*P < 0.05 *vs*. Control or Scramble, Fig. 1A). This data suggests that miR-210 mimic could increase the level of miR-210 in EPCs as well as in EPC-EXs. To determine the time-dependent effects of EXs release, we measured the EXs-concentration at different time points under serum starvation. From Fig. 1B, we can see that the exosome concentration is gradually increased as the time duration is increased (*P < 0.0001 *vs*. 6 h, 12 h, or 24 h). Along with this, we have observed that the exosomes release is reached the maximum at 48 h after serum starvation. Based on that, we used serum starving the EPCs for 48 h for inducing the release of exosomes for the functional studies.

### 3.2. miR-210 released through the exosomes

**Fig. 2.**
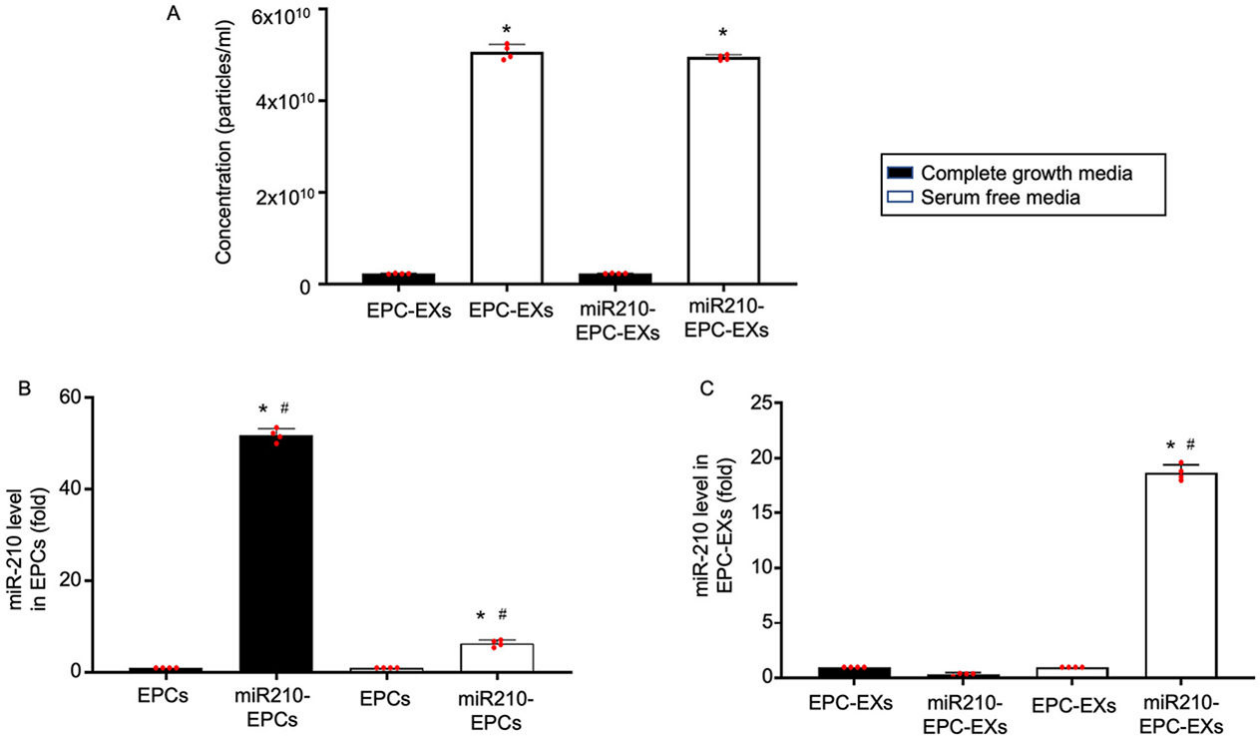
The effect of serum starvation on EXs release and the miR-210 levels in EPCs and EPC-EXs before and after serum starvation. A, The concentration of EXs released from EPCs or miR210-EPCs is significantly increased under serum-free media. *P < 0.05 *vs*. complete growth media. B—C, The level of miR-210 in miR210-EPCs is significantly decreased after serum starvation, while the level of miR-210 in miR210-EPC-EXs is significantly increased after serum starvation. *P < 0.05 *vs*. EPCs or EPC-EXs; ^#^*P* < 0.05 *vs*. complete growth media. Data represent mean — SD, *n* = 4/group.

Moreover, the release of EXs upon culturing EPCs under different situations (serum-free medium or complete medium) was determined by using NTA. We observed that EPCs cultured in a serum-free medium released more exosomes in comparison with cells grown in complete growth media (*P < 0.05 *vs*. complete growth media, Fig. 2A). And there was no difference between the control and transfection groups (*P* > 0.05, EPC-EXs *vs*. miR210-EPC-EXs, Fig. 2A). This data is consistent with previous studies from others and us that serum starvation could promote the release of EXs (Ma et al., 2018; Liu et al., 2017; Garcia et al., 2015). Moreover, the miR-210 levels were evaluated, and the data showed that the miR-210 levels in EPCs grown in serum-free media were much lower than in EPCs grown in complete growth media (^#^*P* < 0.001 *vs*. complete growth media, Fig. 2B). While in the isolated exosomes, it is found that the EPC-EXs isolated from the EPCs cultured in serum-free media had significantly lower miR-210 expression than the EPC-EXs isolated from complete growth media (^#^*P* < 0.001 *vs*. complete growth media, Fig. 2C). This suggests that serum starvation increases the release of EXs from the EPCs and induces the release of miR-210 from EPCs to EPC-EXs.

**Fig. 3.**
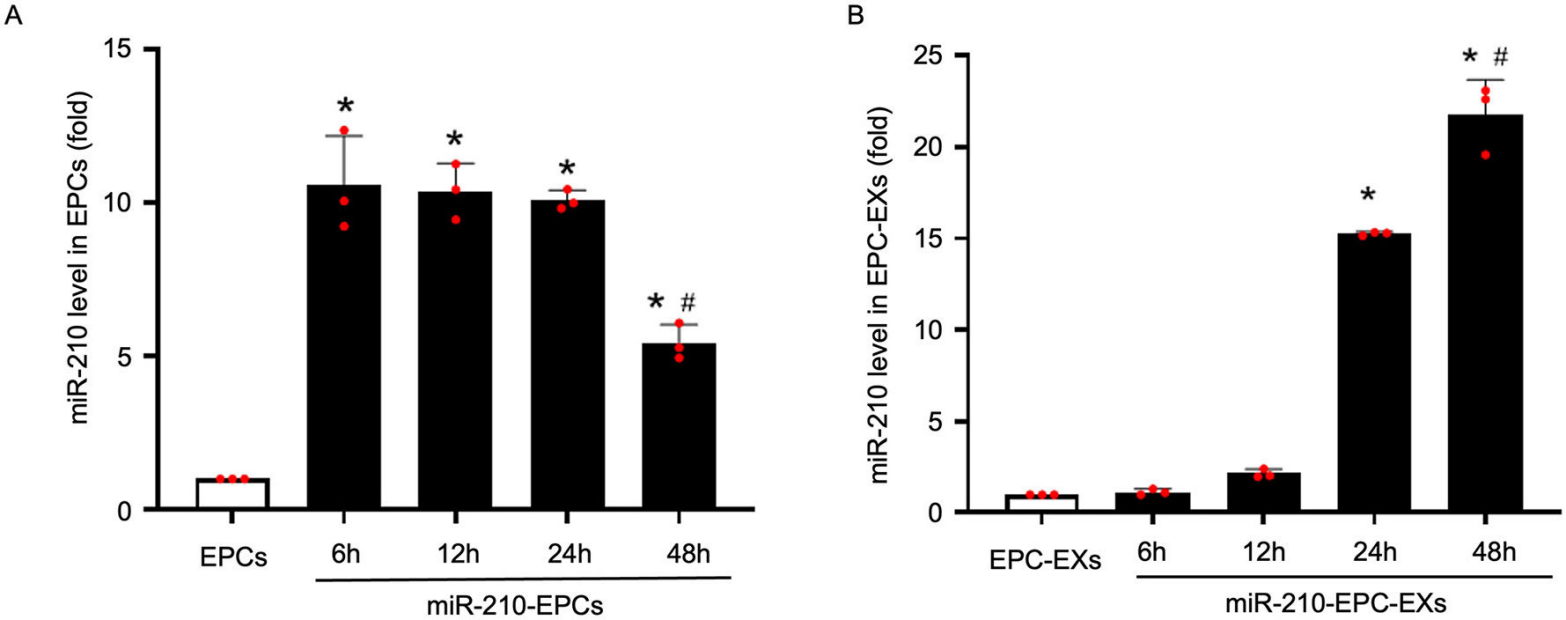
The miR-210 levels in EPCs and EPC-EXs at different time points. The level of miR-210 in miR210-EPCs (A) is decreased; while the level of miR-210 in the miR210-EPC-EXs (B) is increased in a time-dependent manner. *P < 0.05 *vs*. EPCs or EPC-EXs; ^#^*P* < 0.05 *vs*. 6 h, 12 h or 24 h. Data represent mean ± SD, n = 3/group.

Additionally, the miR-210 levels in both EPCs and EXs were also evaluated at corresponding time points. We found that, during the serum starvation, the miR-210 levels in EPCs were decreased with the time duration (Fig. 3, *P < 0.05 *vs*. EPCs or EPC-EXs, ^#^*P* < 0.05 *vs*. 6 h, 12 h or 24 h). Whereas the miR-210 levels in EPC-EXs were increased during the serum starvation duration. This data further confirms that EPC-carried miR-210 was able to be transferred through their released exosomes.

### 3.3. EPC-EXs were uptaken by neurons upon co-culturing

**Fig. 4.**
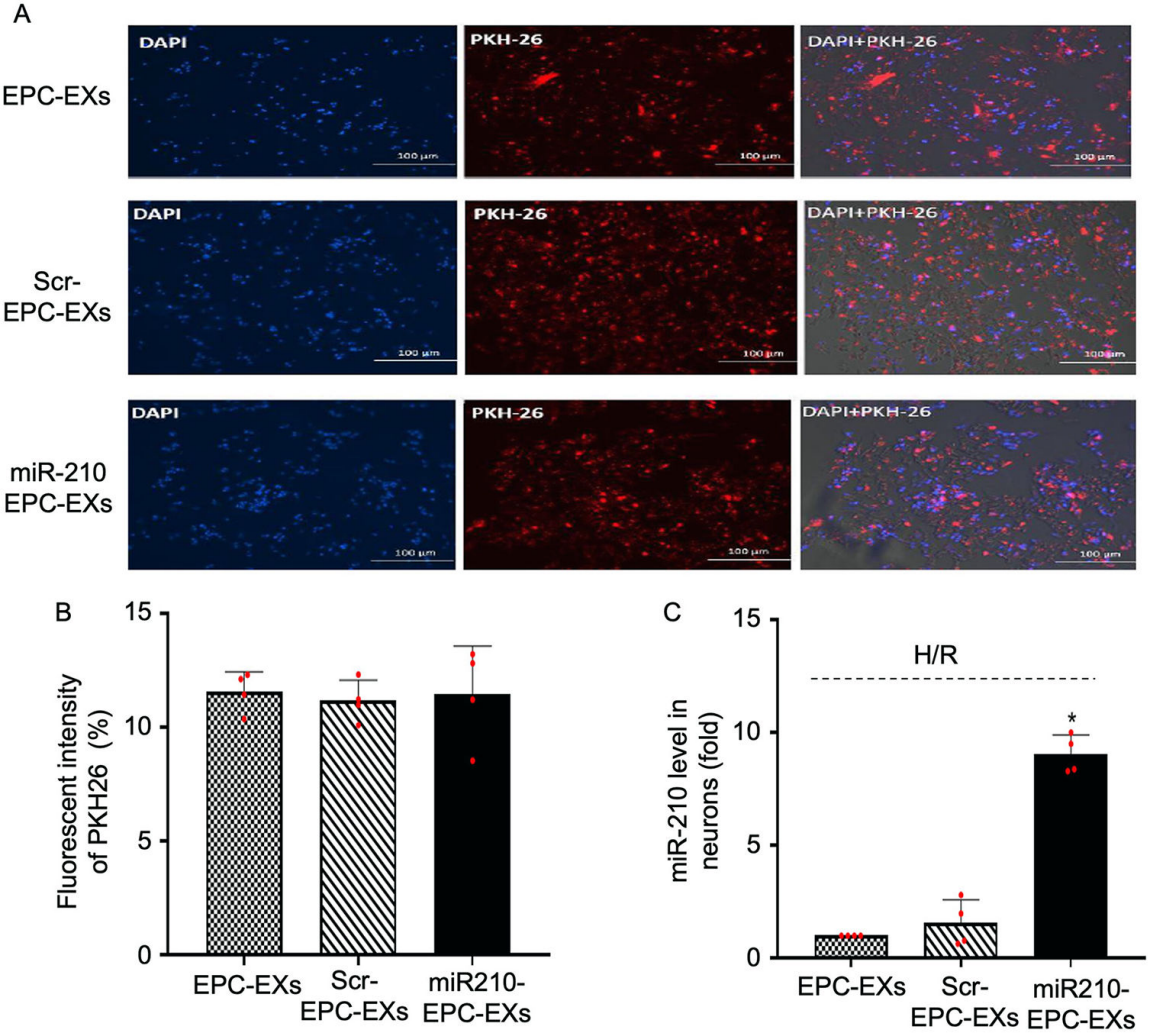
Incorporation of EPC-EXs with neurons after co-culturing. EPC-EXs were labeled with PKH26 (red) to track the incorporation of EPC-EXs with neurons. A, The representative images EPC-EXs incorporation with neurons. Red: PKH26; Blue: DAPI, nucleus; Scale bars: 100 um. B, Summarized data shows that the fluorescent intensity of PKH26 is similar in different groups. C, miR210-EPC-EXs could increase the level of miR-210 in neurons after co-culturing. *P < 0.05 *vs*. EPC-EXs or Scr-EPC-EXs. Scr-EPC-EXs: scramble-EPC-EXs. Data are represented as mean ± SD, n = 4/group.

To determine whether EPC-EXs could be uptaken by neurons, the EPC-EXs were labeled with PKH 26-labeled and we co-incubated with H/R injured neurons for 24 h. After co-incubation, the representative images in Fig. 4A showed that PKH26-labeled EPC-EXs merged with the neurons showing the red fluorescence in the cytoplasm of neurons. Upon measuring the fluorescence intensities (Fig. 4B) in all three groups, there was no significant difference in the fluorescence intensities. This suggests that the incorporation of EPC-EXs to the neurons and the loading of miR-210 into the exosomes did not affect the incorporation rate to the neurons.

### 3.4. miR210-EPC-EXs significantly increased the miR-210 level in neurons upon co-culturing

To confirm the transfer of the miR-210 from miR210-EPC-EXs to the neurons, the level of miR-210 in neurons was evaluated by qRT-PCR after co-incubation. As shown in Fig. 4B, the miR-210 level in the neurons co-cultured with miR210-EPC-EXs was almost 8-folds higher than the neurons co-cultured with EPC-EXs and EPC-EXs from scramble transfected EPCs (*P < 0.05 *vs*. EPC-EXs or Scr-EPC-EXs). This data indicates that miR210-EPC-EXs could deliver miR-210 to neurons after coculturing.

### 3.5. miR210-EPC-EXs were more effective in promoting cell viability in H/R injured neurons than EPC-EXs and scramble-EPC-EXs

**Fig. 5.**
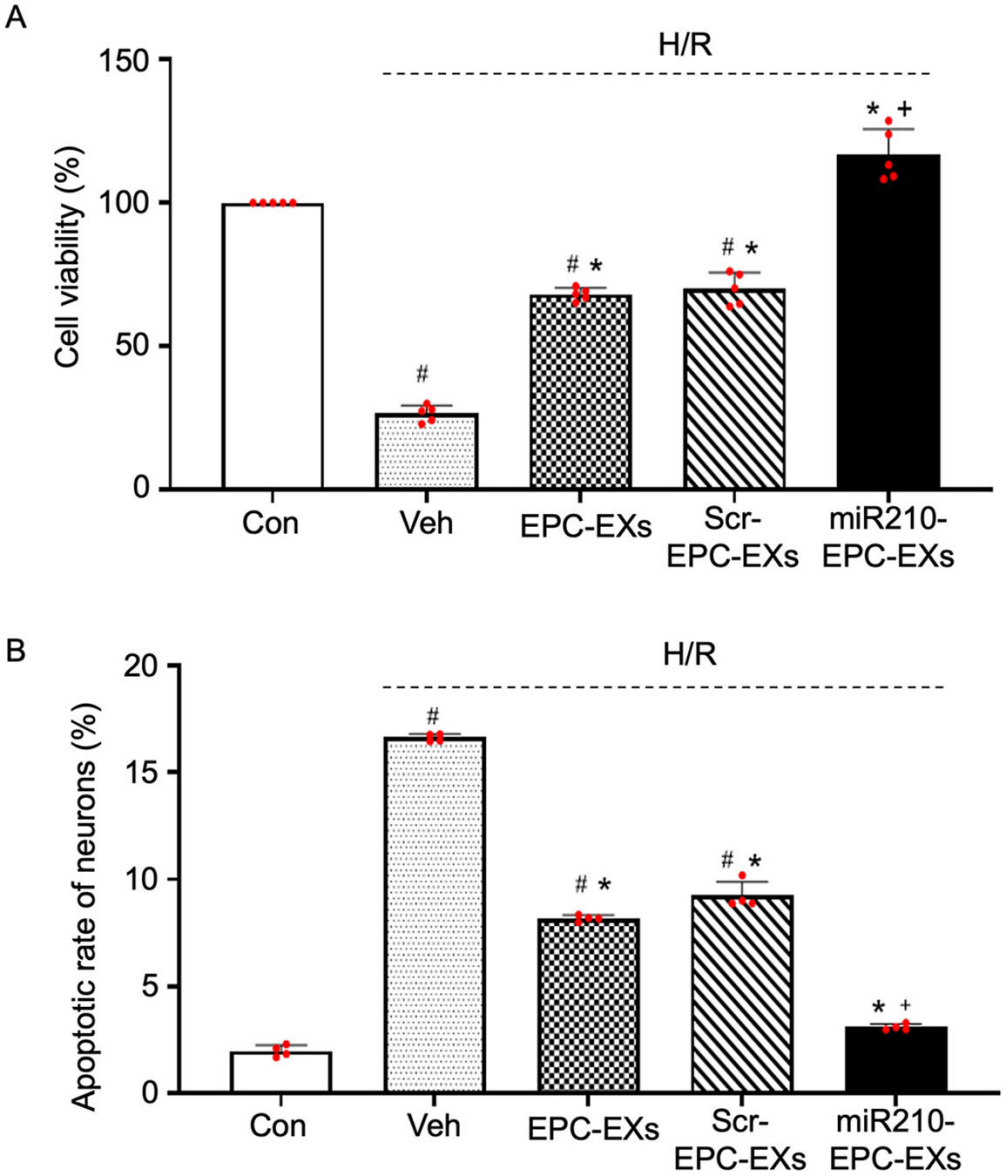
The effects of miR210-EPC-EXs on H/R-induced neurons cell viability and apoptosis. miR-210-EPC-EXs are more effective than EPC-EXs in reducing H/R-induced cell death and apoptosis of neurons. A, Summarized data on the cell viability. Data are represented as mean ± SD, *n* = 5/group B, Summarized data on the apoptotic rate in different groups. Data are represented as mean ± SD, n = 4/group. ^#^*P* < 0.05 *vs*. Con; *P < 0.05 *vs*. Veh; ^+^*P* < 0.05 *vs*. EPC-EXs. Con: control; Veh: vehicle; Scr-EPC-EXs: scramble-EPC-EXs.

To evaluate the function of miR210-EPC-EXs, the effect of miR210-EPC-EXs on H/R injured neurons was determined by a co-culturing experiment. By using MTT assay, we have determined neuronal cell viability. From the MTT data (Fig. 5A), we found that H/R injury significantly reduced the viable cell percentage (^#^*P* < 0.001 *vs*. Con). EPC-EXs and scramble-EPC-EXs have significantly increased the cell viability (*P < 0.001 *vs*. Veh; ^#^*P* < 0.001 *vs*. Con). As we expected, it is observed that miR210-EPC-EXs were more efficient than EPC-EXs and scramble-EPC-EXs in promoting neuronal cell viability (*P < 0.001 *vs*. Veh; ^+^*P* < 0.001 *vs*. EPC-EXs/Scr-EPC-EXs).

### 3.6. miR210-EPC-EXs were more effective than EPC-EXs in reducing H/R induced apoptosis in neurons

To find out whether the increased cell viability was from the reduced apoptosis, the H/R-induced apoptosis of neurons after co-incubation was measured. By using flow cytometry, the apoptotic percentage was determined as Annexin V+/ PI- cells by focusing on early apoptosis. The data (Fig. 5B) showed that H/R injury has significantly elevated the apoptosis percentage in neurons (^#^*P* < 0.001 *vs*. Con), which is partly reduced upon treating with EPC-EXs and scramble-EPC-EXs (*P < 0.001 *vs*. Veh; ^#^*P* < 0.001 *vs*. Con). Moreover, we found that miR210-EPC-EXs have almost completely attenuated the H/R induced apoptosis in neurons (*P < 0.001 vs. Ve; ^+^*P* < 0.001 *vs*. EPC-EXs/Scr-EPC-EXs). The data implies that miR210-EPC-EXs have an enhanced effect on protecting neurons from H/R-induced apoptosis.

### 3.7. Intracellular ROS generation in H/R injured neurons was attenuated more efficiently by miR210-EPC-EXs than EPC-EXs and scramble-EPC-EXs

**Fig. 6.**
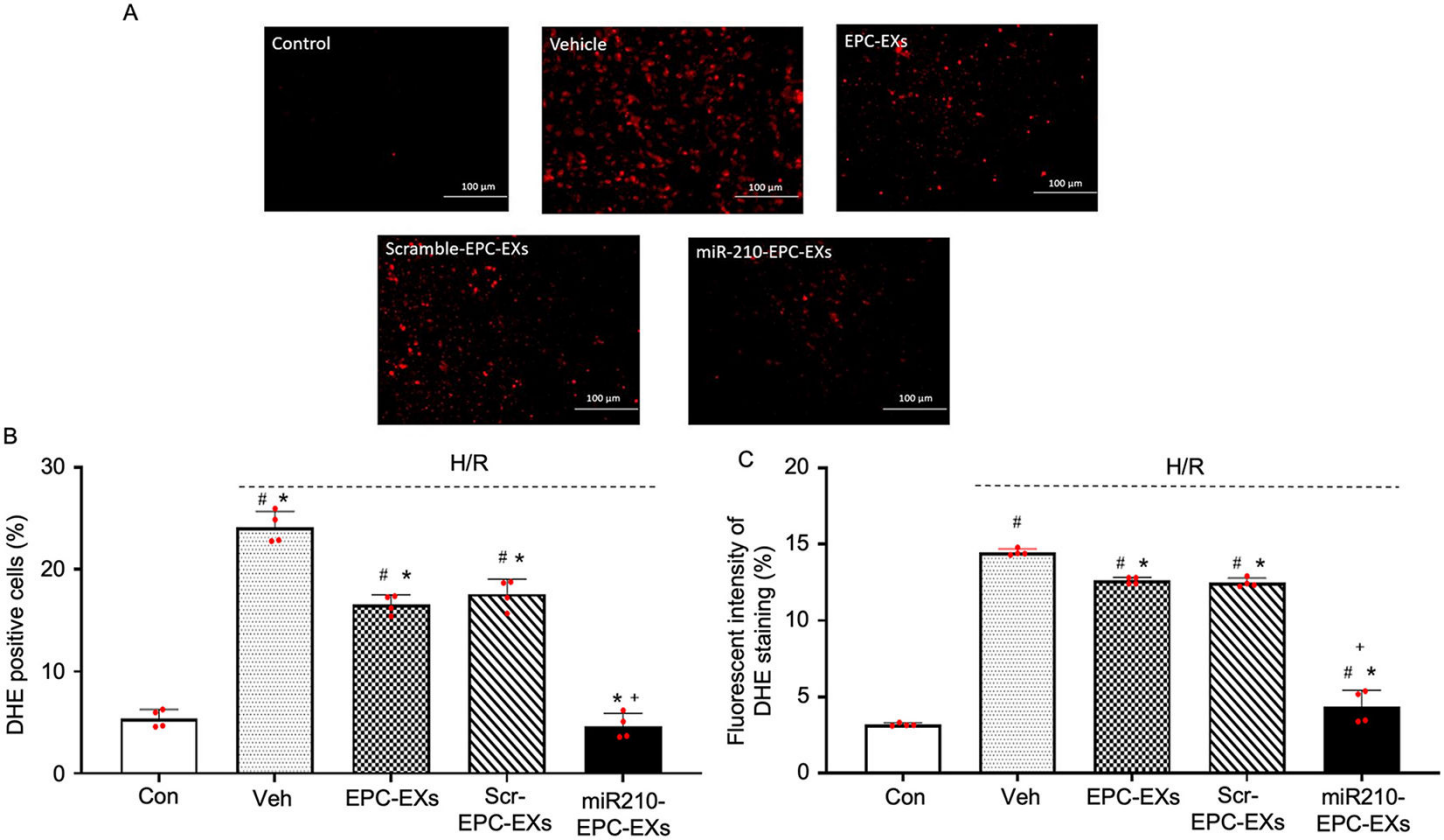
The effects of miR210-EPC-EXs on H/R-induced oxidative stress in neurons. miR-210-EPC-EXs are more effective than EPC-EXs in attenuating H/R-induced intracellular ROS generation in neurons. A, Representative images of DHE staining. Red: DHE. Scale bars: 100 um. B, Summarized data on the fluorescent intensity of DHE staining. C, Summarized data on the DHE positive cells by flow cytometry. ^#^*P* < 0.05 *vs*. Con; *P < 0.05 *vs*. Veh; ^+^*P* < 0.05 *vs*. EPC-EXs. ROS: reactive oxide species; Con: control; Veh: vehicle; Scr-EPC-EXs: scramble-EPC-EXs. Data are represented as mean ± SD, n = 4/group.

Elevated intracellular ROS levels are one of the most important factors for cell injury. To verify whether miR210-EPC-EXs have elevated protective effects in reducing ROS levels in H/R neurons over EPC-EXs/scramble-EPC-EXs, we perform intracellular ROS generation assay 24 h. after co-culturing. In presence of ROS, the superoxide indicator-DHE produces red fluorescence in the neurons (Fig. 6A). The DHE-positive cells were detected using flow cytometry (Fig. 6B), the images were captured by fluorescence microscope (Fig. 6A) and the fluorescence intensities were determined for each group (Fig. 6C). The data (Fig. 6B, and C) showed that H/R injury has significantly elevated the ROS levels in neurons (^#^*P* < 0.001 *vs*. Con), which is reduced to a certain extent upon treating with EPC-EXs and scramble-EPC-EXs (*P < 0.001 *vs*. Veh; ^#^*P* < 0.001 *vs*. Con). Moreover, we found that miR210-EPC-EXs have profoundly reduced the ROS levels in H/R injured neurons (*P < 0.001 *vs*. Veh; ^+^*P* < 0.001 *vs*. EPC-EXs/Scr-EPC-EXs). This data demonstrates that miR210-EPC-EXs are more capable than EPC-EXs and scramble-EPC-EXs in reducing ROS levels in H/R injured neurons.

### 3.8. miR210-EPC-EXs protected neurons from H/R induced injury through BDNF/TrkB and Nox2/Nox4 pathways

**Fig. 7.**
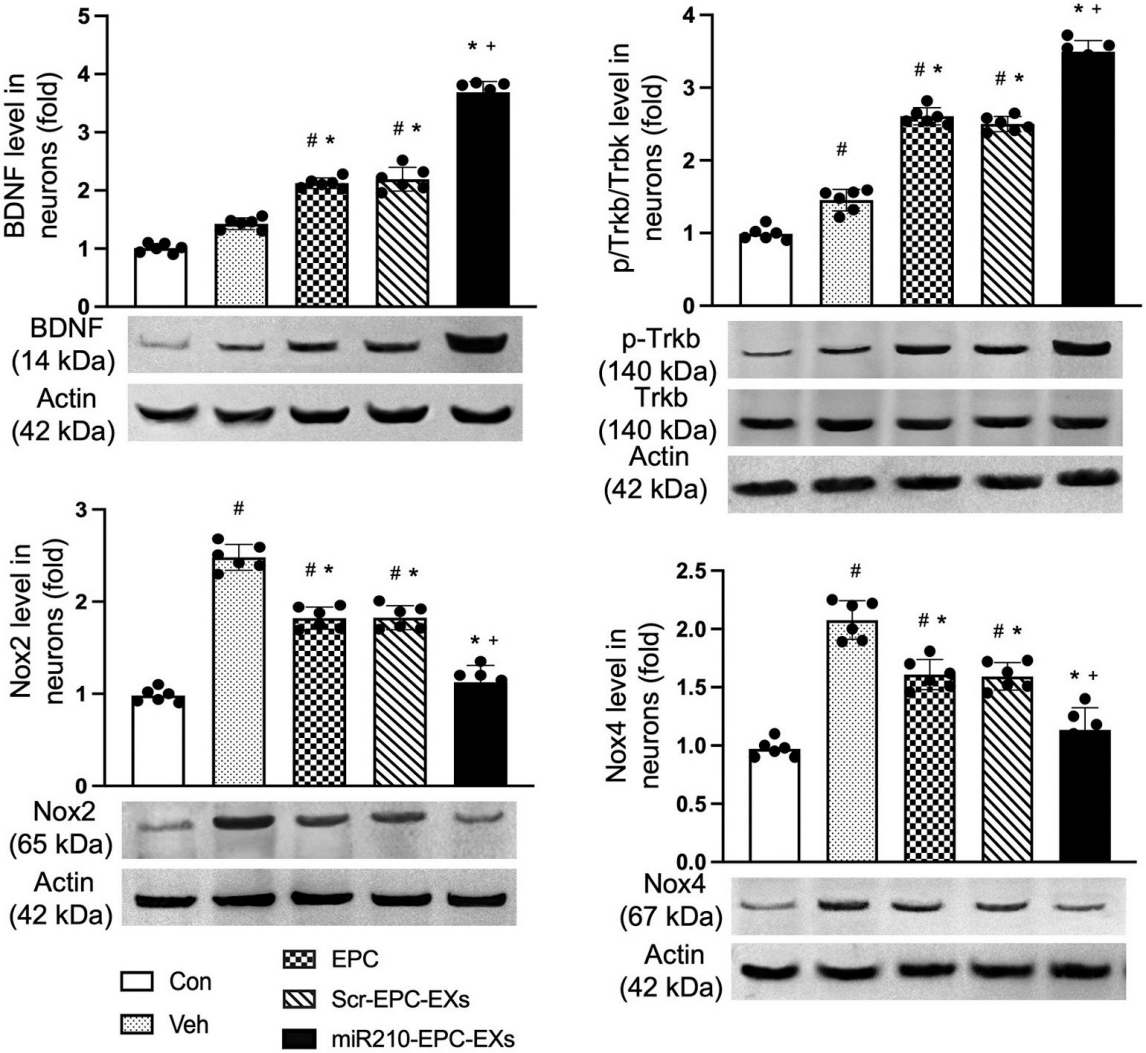
The mechanisms underlying the beneficial effects of miR210-EPC-EXs on H/R-induced neuron injury. The protein levels of BDNF, p-TrkB/TrkB, and Nox2/Nox 4 were determined in different treatment groups. miR210-EPC-EXs were more effective in upregulating BDNF and p-TrkB/TrkB expression while downregulating Nox2 and Nox4 expression in neurons under H/R. ^#^*P* < 0.05 *vs*. Con; *P < 0.05 *vs*. Veh; +*P* < 0.05 vs. EPC-EXs. Con: control; Veh: vehicle; Scr-EPC-EXs: scramble-EPC-EXs. Data are represented as mean ± SD, *n* = 6/group.

To explore the underlying mechanisms that are responsible for the protective effects of miR210-EPC-EXs on H/R injured neurons, the protein expression of BDNF, p-TrkB/TrkB, and Nox2/Nox4 were evaluated in neurons after different treatments. The Western blot data showed that H/R downregulated the expression of BDNF and p-TrkB/TrkB, but upregulated the expression of Nox2/Nox4 (Fig. 7, ^#^*P* < 0.001 *vs*. Con). More importantly, we found that miR210-EPC-EXs could reverse these effects and had better efficacy than EPC-EXs (Fig. 7, *P < 0.001 *vs*. Veh; ^+^*P* < 0.001 *vs*. EPC-EXs/Scr-EPC-EXs). This could be explained that the miR210-EPC-EXs protected neurons from H/R-induced cell death and oxidative stress through BDNF/TrkB and Nox2/Nox4 pathways, respectively.

## 4. Discussion

In the present study, we investigated the beneficial effects of EPC-EXs on protecting the neurons from H/R-injury by reducing apoptosis, oxidative stress and by increasing cell viability. Interestingly, we also found that the loading of miR-210 in EPC-EXs boosted these protective effects.

We have reproduced the H/R injury model in neurons to investigate the protective effects of EPC-EXs and miR210-EPC-EXs against H/R injury (Ma et al., 2018; Wang et al., 2020b). As we know, brain cells require a sufficient amount of oxygen and glucose to generate ATP and maintain membrane potential for normal functioning (Benjamin et al., 2017; Onwuekwe and Ezeala-Adikaibe, 2012). During ischemic stroke, as the artery that supplies blood to the brain is blocked, the neurons do not receive blood supply properly and so they are oxygen-deprived. This results in ATP level depletion and rises the intracellular calcium levels (Hui et al., 2022) which cause mitochondrial injury, free radical generation, and eventually leads to cell death by damaging the nucleus and cell membrane (Hui et al., 2022; Saver, 2006). As expected, we confirmed the H/R injury as evidenced by increasing neuronal apoptosis, oxidative stress and eventually reducing the viability of neurons.

EPCs have been shown to provide beneficial effects on ischemic stroke (Esquiva et al., 2018). Our previous work has reported that EPCs transfusion will reduce cerebral ischemic damage (Chen et al., 2011; Chen et al., 2013). A study by Liao S et al. demonstrated that EPCs can protect the neurons from ischemic injury by repairing secretory functions and vascular endothelium (Liao et al., 2017). Besides this EPCs are also known for their angiogenesis, wound healing ability, and tissue regeneration (Asahara, 2007). The mechanism underlying the therapeutic effects of EPCs could be from their differentiation to ECs to form new vessels, and also from their released growth factors which promote angiogenesis and neurogenesis (Asahara, 2007). Most recently, research suggests that their released EXs could be one of the important contributors to their beneficial effects (Boriachek et al., 2018).

EXs are the smallest extracellular vesicles, these lipid bilayer vesicles carry mRNA, miRNA, DNA, lipids, proteins, and some components from the Golgi apparatus, mitochondria, endoplasmic reticulum, and nucleus (Yanez-Mo et al., 2015). They are formed by the inward budding of endosomes and later released into the extracellular space upon fusing with the plasma membrane (Crenshaw et al., 2018; Raposo and Stoor-vogel, 2013). They communicate with other cells by docking and fusing. They play an important role in transferring information from one cell to another also referred to as intercellular communication (Rahmati et al., 2020). Along with this EXs are known to possess a longer circulating half-life and they do not have a risk of transplant rejection and aneuploidy all these postulates made exosomes the safest therapeutic tools (Boriachek et al., 2018). EXs derived from a variety of cell types such as embryonic stem cells, induced pluripotent stem cells, adult stem cells such as mesenchymal stromal cells, human umbilical cord blood cells, neural stem cells, and EPCs have been employed to treat ischemic stroke and other cerebrovascular diseases in pre-clinical studies (Boriachek et al., 2018). Our previous studies found that post-stroke damage can be alleviated by infusing EPC-EXs (Wang et al., 2020a). Ma, Xiaotang, et al. found that EPC-EXs can protect endothelial cells from hypoxia and reoxygenation injury by protecting mitochondrial functionality (Ma et al., 2018).

In the present study, we first determined the release pattern of EXs by culturing EPCs in different conditions. Previous studies have shown that EXs release could be promoted upon different stimuli (Gu et al., 2014; Wang et al., 2013a). Here, we compared the EXs release pattern upon culturing EPCs in complete growth media and serum-free conditions. Results stated that EXs release is higher under stress *i.e*., serum starvation (Fig. 2). This is consistent with the previous studies (Garcia et al., 2015; Gu et al., 2014). Moreover, we performed the time-dependent effects of EXs release from EPCs. The data in Fig. 1 explains that EXs release is gradually increased and reached the peak at 48 h after serum starvation. Basing this, we used 48 h as the serum starvation duration to achieve maximum exosomal concentration in the present study.

As we have discussed previously, EXs are lipid bilayer vesicles that can carry mRNA, miRNA, DNA, lipids, proteins and have an important role in intercellular communication (Boriachek et al., 2018; Rahmati et al., 2020). The function of EXs is highly related to their carried molecules, they are known to convey the benefit of one cell to another cell *via* miRNAs (Rahmati et al., 2020). It is known that miR-210 is involved in cell protection with anti-apoptotic and anti-oxidant properties by reducing ROS production (Jiang et al., 2015; Wang et al., 2013b; Mutharasan et al., 2011). To date, it has been demonstrated that miR-210 not only influences cell survival by targeting apoptosis genes (caspase, Bcl-2, Bax, *etc*.) but also that it slows down mitochondrial respiration to increase cell survival (Kim et al., 2009; Zhu and Fan, 2012). However, it has been reported that the miR-210 level is significantly decreased in stroke patients at 7 and 14 days after stroke onset and that the circulating miR-210 level is positively related to the outcome of stroke patients (Zeng et al., 2011). Lentivirus-mediated overexpression of miR-210 has been shown to improve long-term outcomes after focal cerebral ischemia in mice (Zeng et al., 2016). Of note, we have previously shown that NPC-EXs alleviate endothelial oxidative stress and dysfunction through the miR-210 downstream Nox2 and vascular endothelial growth factor pathways (Liu et al., 2017). miR210-EPC-EXs can protect endothelial cells from hypoxia and reoxygenation injury by protecting mitochondrial function (Ma et al., 2018). Considering these postulates of miR-210, in this study we further evaluated the role of miR-210 in the function of EPC-EXs on protecting neurons from H/R-induced injury. In order to raise the miR-210 expression in EPC-EXs, we have transfected the EPCs with miR-210 mimic. The rise in miR-210 level in EPCs is confirmed by qPCR (Fig. 1). We also demonstrated the flow of miR-210 release from cells to exosomes, Fig. 3 illustrates that with the increase in the duration of serum starvation in EPCs, the miR-210 levels are being decreased. Whereas in EXs with the increased serum starvation duration there is a rise in both exosome release and miR-210 levels. This explains that as the duration of serum starvation is increasing the cells tend to release more exosomes and tend to transfer more miR-210, that is the reason we have observed a reduction in miR-210 levels in cells and a rise in miR-210 levels in exosomes.

Meanwhile, upon co-incubating PKH 26-labeled EPC-EXs with neurons for 24 h. we found that (Fig. 4) the EPC-EXs have successfully incorporated into the neurons. And by comparing the fluorescence intensities of the images it was clear that the loading of miR-210 into the exosomes did not affect the incorporation rate to the neurons (Fig. 4B). In addition to this, we have quantified miR-210 levels in neurons. The data (Fig. 4C) explains that the miR-210 level in the neurons co-cultured with miR-210 EPC-EXs is significantly higher than the neurons co-cultured with EXs that are isolated from control and scramble transfected groups. This suggests that miR-210- EPC-EXs can deliver miR-210 to neurons after coculturing. That is the reason we have observed a reduction in miR-210 levels in cells and a rise in miR-210 levels in exosomes. Upon evaluating the functional effects of EPC-EXs on H/R injured neurons, we found that EPC-EXs have significantly reduced the apoptosis percentage (Fig. 5), ROS generation (Fig. 6), and finally increased the viability of neurons (Fig. 5). In addition to this, we found that miR210-EPC-EXs are more efficient than EPC-EXs in reducing apoptosis percentage, oxidative stress, and increasing cell viability of H/R injured neurons.

For the mechanism underlying the protective effects of miR210-EPC-EXs on the neurons, we measured the protein levels related to the miR210 downstream pathway in the neurons after co-incubation. Among the functional molecules in the central nervous system (CNS), one of the important groups is the neurotrophins, which are nerve growth factor (NGF)-related gene family molecules. Neurotrophins include NGF, BDNF, neurotrophin 3 (NT-3), and NT-4/5 in the mammal. Among neurotrophins and their receptors, BDNF and TrkB are enriched in the CNS. The BDNF-TrkB signaling pathway regulates a wide variety of neuronal functions, such as cell survival, migration, outgrowth of axons and dendrites, synaptogenesis, synaptic transmission, and remodeling of synapses. We found that the upregulation of the BDNF/TrkB pathway in neurons after the coincubation with miR210-EPC-EXs might be responsible for the enhanced cell survival rate. For the reduced ROS production, we measured the level of Nox2/4 levels which is a major factor regulating oxidative stress. As we expected, the level Nox2/4 is decreased in the neurons after the treatment of miR210-EPC-EXs, which could be the mechanism contributing to the antioxidant effects of miR210-EPC-EXs.

In summary, our study demonstrates that under serum starvation, EPCs tend to release more EXs and the release of EXs is further increased with the duration of stress. During EXs release, the miR-210 from the EPCs is transferred to EXs. EPC-EXs can be uptaken by the neurons and deliver the miR-210 to neurons by EPC-EXs. Our results demonstrate that miR-210 loading can enhance the protective effects of EPC-EXs on H/R induced elevated apoptosis, oxidative stress and decreased the viability of neurons. The BDNF/TrkB and Nox2/Nox4 pathways could be the underlying mechanism for the protective effects of miR210-EPC-EXs.

## Data Availability

Data will be made available on request.

## Abbreviations:

EXs: exosomes
EPCs: endothelial progenitor cells
H/R: hypoxia/reoxygenation
miR: microRNA
EPC-EXs: endothelial progenitor cell-released exosomes
miR210-EPC-EXs: miR1210overexpressed EPC-EXs
tPA: tissue Plasminogen Activator
ECs: endothelial cells
NPCs: neural progenitor cells
NTA: nanotracking Analysis
ROS: reactive oxygen species
DHE: dihydroethidium
BDNF: brain-derived neurotrophic factor
TrkB: Tropomyosin receptor kinase B
Nox-2: NADPH oxidase 2
